# Effects of pre-existing anti-adenovirus antibodies on transgene expression levels and therapeutic efficacies of arming oncolytic adenovirus

**DOI:** 10.1038/s41598-022-26030-3

**Published:** 2022-12-13

**Authors:** Ryosuke Ono, Fumitaka Nishimae, Takuro Wakida, Fuminori Sakurai, Hiroyuki Mizuguchi

**Affiliations:** 1 Laboratory of Biochemistry and Molecular Biology, Graduate School of Pharmaceutical Sciences, Osaka University, 1-6 Yamadaoka, Suita-City, Osaka Japan; 2 Laboratory of Functional Organoid for Drug Discovery, Center for Drug Discovery Resources Research, National Institutes of Biomedical Innovation, Health and Nutrition, 7-6-8, Saito-Asagi, Ibaraki-City, Osaka Japan; 3 Global Center for Medical Engineering and Informatics, Osaka University, 1-6 Yamadaoka, Suita-City, Osaka Japan; 4 Integrated Frontier Research for Medical Science Division, Institute for Open and Transdisciplinary Research Initiatives (OTRI), Osaka University, 1-6 Yamadaoka, Suita-City, Osaka Japan; 5 Center for Infectious Disease Education and Research (CiDER), Osaka University, 1-6 Yamadaoka, Suita-City, Osaka Japan

**Keywords:** Virology, Drug development, Gene delivery, Gene therapy, Cancer therapy

## Abstract

Oncolytic adenoviruses (OAds), most of which are based on species C human adenovirus serotype 5 (Ad5) (OAd5), have recently received much attention as potential anticancer agents. High seroprevalence of anti-Ad5 neutralizing antibodies is a major hurdle for Ad5-based gene therapy. However, the impacts of anti-Ad5 neutralizing antibodies on OAd5-mediated transgene expression in the tumor and antitumor effects remain to be fully elucidated. In this study, we examined the impact of anti-Ad5 neutralizing antibodies on the OAd5-mediated antitumor effects and OAd5-mediated transgene expression. The luciferase expression of OAd-tAIB-Luc, which contains the cytomegalovirus promoter-driven luciferase gene, was inhibited in human cultured cells in the presence of human serum. Although the inhibitory effects of human serum possessing the low anti-Ad5 neutralizing antibody titers were overcome by long-term infection, the in vitro tumor cell lysis activities of OAd-tAIB-Luc were entirely attenuated by human serum containing the high titers of anti-Ad5 neutralizing antibodies. OAd-tAIB-Luc-mediated luciferase expression in the subcutaneous tumors 3 days after administration and tumor growth suppression levels following intratumoral administration were significantly lower in mice possessing the high titers of anti-Ad5 neutralizing antibodies, compared to those in control mice. These results suggested that pre-existing anti-Ad5 antibodies attenuated both transgene expression and potential antitumor effects of OAd5 following intratumoral administration.

## Introduction

Recently, virotherapy using oncolytic viruses (OVs) has shown promising results and thus has been gaining more attention^1,2^. OVs selectively infect and kill tumor cells without apparent toxicity to normal cells. OVs have several advantages. One of the major advantages is that the antitumor effects of OVs can be enhanced by inserting a therapeutic transgene expression cassette in the virus genome, a strategy known as arming^3–6^. Several types of therapeutic genes, including cytokine genes and tumor suppressor genes, can be incorporated into the OV genome in this manner. Hence, armed OVs show promising antitumor effects by both tumor-specific replication-mediated tumor cell lysis and the therapeutic transgene expression^7–9^.

Among the various types of OVs, oncolytic adenovirus (Ad) (OAd) is one of the most widely used^10^. Tumor-selective replication of OAd is mediated by tumor-specific promoter-derived E1 gene expression^11^ or genetic modification of the E1 gene, which encodes the proteins required for Ad self-replication, such as the lack of the E1B55k gene^12^ and the mutation in the E1A gene^13^. Almost all OAds are composed of species C human Ad serotype 5 (Ad5) (OAd5). OAd5 shows efficient tumor cell lysis activity^14–16^. In addition, OAd5 can be easily modified by genetic engineering and has a relatively large capacity for accommodating therapeutic transgenes. However, anti-Ad5 neutralizing antibodies are a major hurdle to the therapeutic use of OAd5. More than 60% of adults have anti-Ad5 neutralizing antibodies due to natural exposure to Ad5^17–23^. There is thus a concern that pre-existing anti-Ad5 neutralizing antibodies can attenuate transgene expression in the tumors and the antitumor effects of OAd5. However, the impact of pre-existing anti-Ad5 neutralizing antibodies on OAd5-mediated transgene expression and antitumor effects in tumor-bearing hosts has not been fully evaluated because there are no appropriate mouse models in which the impact of anti-Ad5 neutralizing antibodies on both OAd5-meditated transgene expression and OAd5-meditated antitumor effects can be simultaneously evaluated. Since mouse cells are not permissive to human Ad replication, attempts to establish such a model have involved transplantation of human tumor cells to immune-incompetent mice; however, almost no anti-Ad5 antibodies are produced after immunization with an Ad5 in immune-incompetent mice. The Syrian hamster is available as an immune-competent model for OAd research since Syrian hamster cells are permissive to human Ad infection^24^. However, only several types of Syrian hamster tumor cell lines are available for OAd studies. Moreover, it is unclear whether the hTERT promoter efficiently works in Syrian hamster tumor cells at levels comparable to human tumor cells.

In this study, we produced human subcutaneous tumor-bearing nude mice possessing anti-Ad5 antibodies by injecting mouse anti-Ad5 serum in nude mice. This model can evaluate the antitumor effects of OAd against human tumors in mice possessing anti-Ad5 neutralizing antibodies. Titers of anti-Ad5 neutralizing antibodies in the nude mice after injection of mouse anti-Ad5 serum were comparable to those in human serum. We then examined the in vivo inhibitory effects of pre-existing anti-Ad5 neutralizing antibodies on the transgene expression levels and antitumor effects of OAd5 following intratumoral administration.

## Results

### The effects of human serum on OAd5-mediated transgene expression in the cultured cells

**Figure 1:**
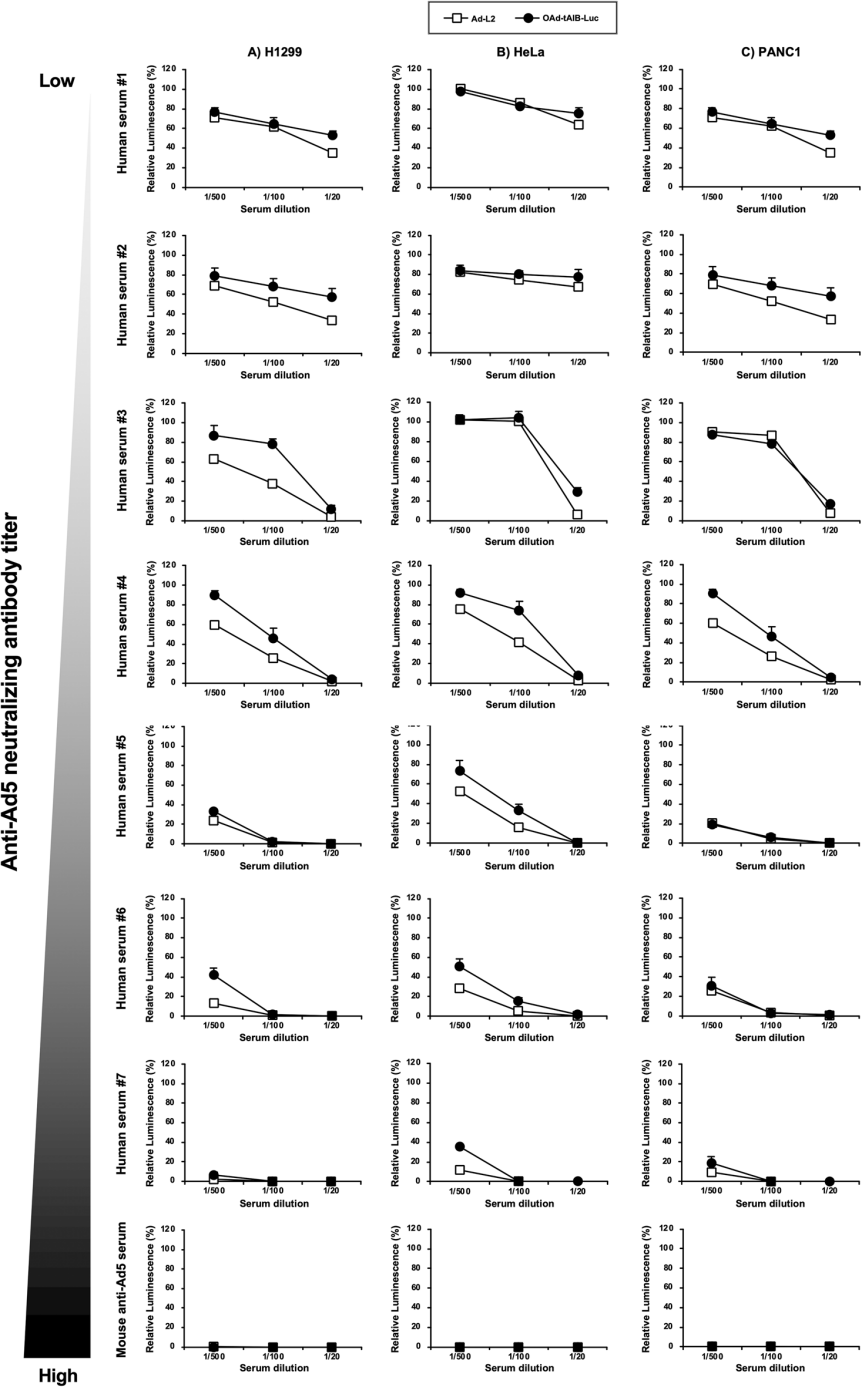
In vitro luciferase expression of Ad-L2 and OAd-tAIB-Luc in the presence of mouse and human serum. Ad-L2 and OAd-tAIB-Luc were pre-incubated with diluted mouse and human serum followed by adding to (**A**) H1299, (**B**) HeLa, and (**C**) PANC-1 cells at 200 VP/cell. Luciferase activities were measured 3 days after virus treatment. The luminescence levels in the cells without pre-incubation with human serum were normalized to 100%. These data are expressed as the means ± S.D. (n = 4).

First, in order to examine the in vitro transgene expression levels induced by firefly luciferase-expressing OAd5, OAd-tAIB-Luc, in the presence of mouse and human serum, OAd-tAIB-Luc was added to the cells after pre-incubation with mouse and human serum (Fig. 1). OAd-tAIB-Luc has the human telomerase reverse transcriptase (hTERT) promoter-driven E1A gene linked with the E1B gene by the internal ribosome entry site (tAIB) and the cytomegalovirus (CMV) promoter-driven luciferase gene cassette^25^. The previous study demonstrated that OAd containing the same E1 gene expression cassette mediates no apparent cytotoxicity in normal cells^26^. A replication-incompetent Ad vector expressing firefly luciferase, Ad-L2, was used as a control. The luciferase expression of Ad-L2 was about tenfold higher than OAd-tAIB-Luc in the human tumor cell lines (Supplementary Fig. S1). Whereas the luciferase expression levels were strongly decreased for both Ad-L2 and OAd-tAIB-Luc in correlation with the anti-Ad5 neutralizing antibody titers in the serum, the human serum suppressed the luciferase expression of Ad-L2 to a slightly higher degree than that of OAd-tAIB-Luc. Similar results were found in the three types of human tumor cells. These human tumor cell lines were relatively permissive to Ad vector-mediated transduction. These data indicated that anti-Ad5 neutralizing antibodies attenuated not only Ad vector-mediated, but also OAd-mediated transgene expression in cultured cells, although OAd-mediated transgene expression was less effectively inhibited by human serum, compared with Ad vector-mediated transgene expression.

### The effects of human serum on OAd-mediated in vitro tumor cell lysis activities

**Figure 2:**
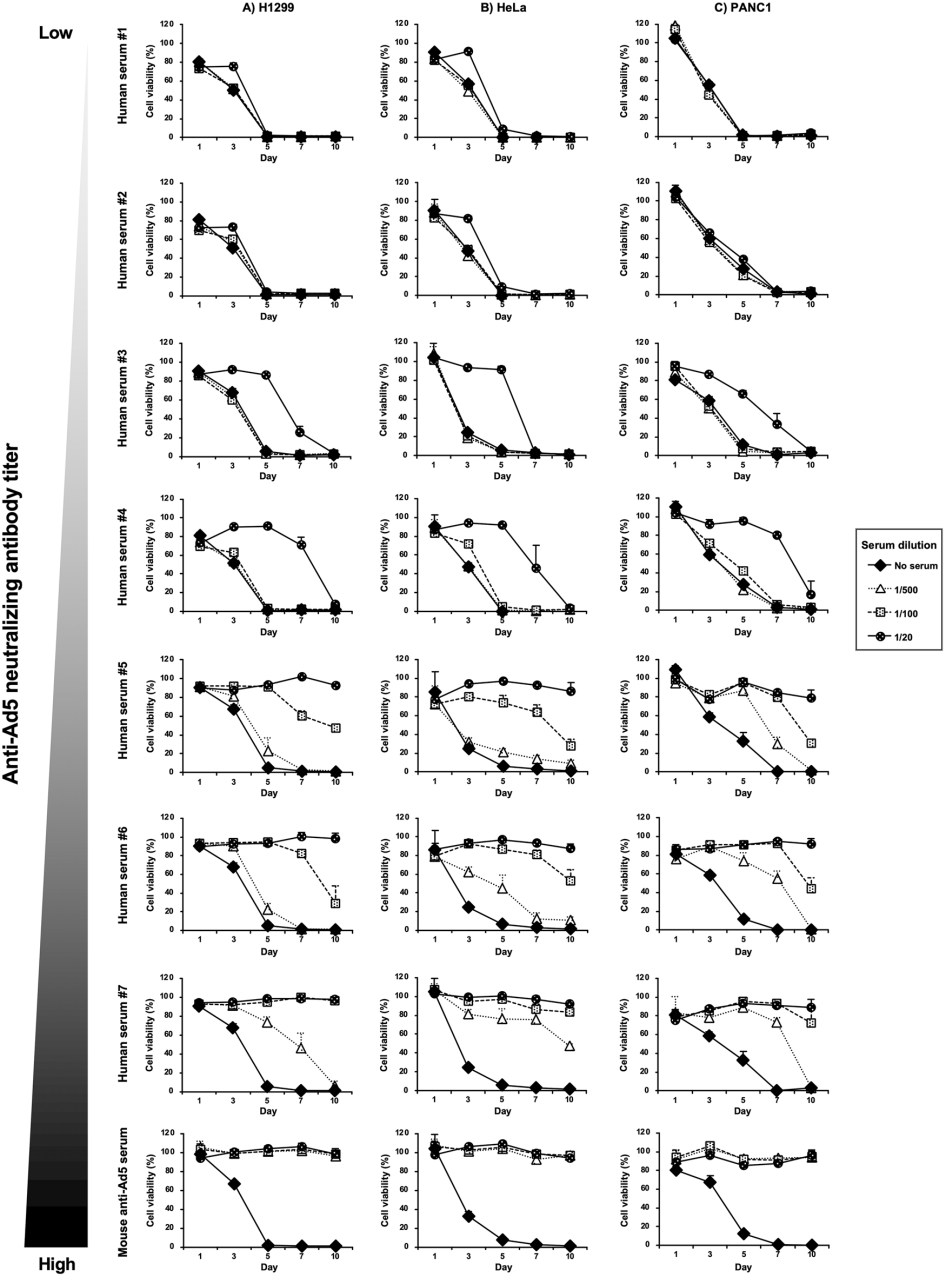
In vitro tumor cell lysis activities of OAd-tAIB-Luc in the presence of mouse and human serum. OAd-tAIB-Luc was pre-incubated with mouse and human serum, followed by infection with (**A**) H1299, (**B**) HeLa, and (**C**) PANC-1 cells at 200 VP/cell. At the indicated time points, cell viabilities were determined by WST-8 assay. The viability of the mock-infected group was normalized to 100%. These data are expressed as the means ± S.D. (n = 4).

Next, tumor cell viabilities were measured at the indicated time points to examine the impact of mouse and human serum on the in vitro tumor cell lysis activities of OAd-tAIB-Luc (Fig. 2). The tumor cell viabilities gradually declined following infection with OAd-tAIB-Luc in all human tumor cells in the absence of the serum. The serum containing the high titers of anti-Ad5 neutralizing antibodies significantly inhibited the in vitro tumor cell lysis activities of OAd-tAIB-Luc after short and prolonged-term infection (Fig. 2, Supplementary Figs. S2 and S3). On the other hand, in the presence of human serum containing the low titers of anti-Ad5 neutralizing antibodies, the viabilities of all tumor cells largely declined after a 10-day incubation, although the in vitro tumor cell lysis activities of OAd-tAIB-Luc were inhibited at early time points. These data indicated that although the long-term infection overcame the inhibitory effects of human serum containing low titers of anti-Ad5 neutralizing antibodies, OAd5-mediated in vitro tumor cell lysis activities were entirely hindered by high titers of anti-Ad5 neutralizing antibodies.

The effects of anti-Ad5 neutralizing antibodies on OAd5-mediated transgene expression and tumor growth suppression in tumor-bearing mice.

**Figure 3:**
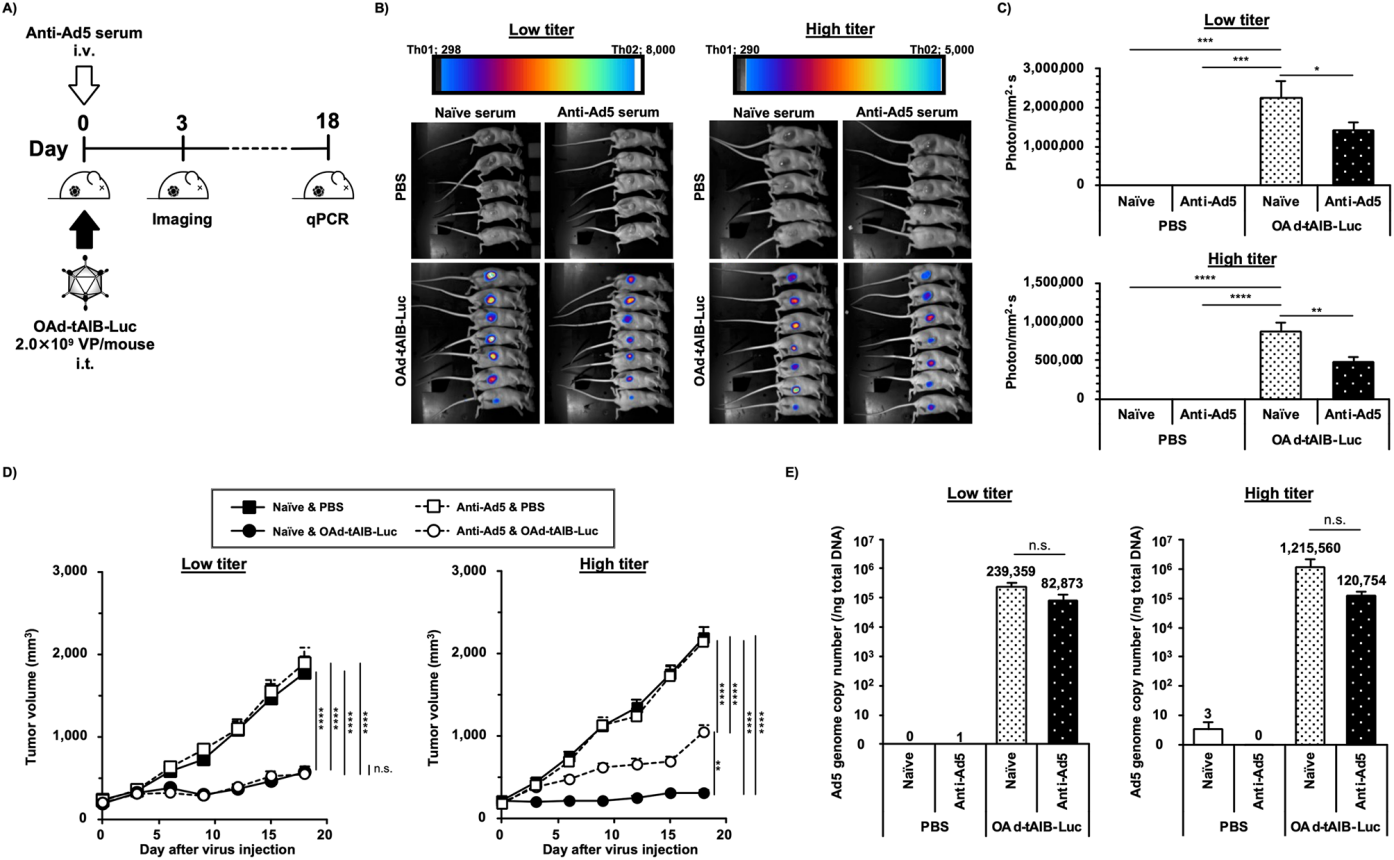
In vivo suppressive effects of anti-Ad5 neutralizing antibodies on the transgene expression in the tumor and antitumor effects of OAd-tAIB-Luc. (**A**) Schematic diagram of experimental schedule. BALB/c nu/nu mice bearing H1299 tumors were intratumorally administered OAd-tAIB-Luc at 2.0 × 10^9^ VP/mouse 1 h after intravenous administration of the low- or high-dose of mouse anti-Ad5 serum. (**B**) In vivo imaging of firefly luciferase expression in the tumor following OAd-tAIB-Luc administration. D-luciferin solution was intravenously injected at 2.0 mg/mouse 3 days after virus administration. (**C**) Firefly luciferase luminescence intensities in the tumors following OAd-tAIB-Luc administration. The luminescence intensities in the tumors shown in (**B**) were quantified. One-way ANOVA followed by Dunnett’s multiple comparisons test was performed. *; *P* < 0.05, **; *P* < 0.01, ***; *P* < 0.001, ****; *P* < 0.0001 (vs. OAd-tAIB-Luc and naïve serum-pre-injected group). Data are expressed as the means ± S.E. (PBS; n = 5, OAd-tAIB-Luc; n = 7). (**D**) Tumor growth following OAd-tAIB-Luc administration. The tumor volumes were measured every 3 days after administration. Two-way ANOVA with Bonferroni's multiple comparisons post hoc test was used for statistical analysis. **; *P* < 0.01, ****; *P* < 0.0001, n.s.; not significant. Tumor volume is expressed as the mean tumor volume ± S.E. (PBS; n = 5, OAd-tAIB-Luc; n = 7). (**E**) Ad genome copy numbers in the tumors on day 18 after administration. Total DNA was isolated from the tumors on day 18 after OAd-tAIB-Luc administration, followed by real-time PCR analysis. One-way ANOVA followed by Dunnett’s multiple comparisons test was performed. n.s.; not significant (vs. OAd-tAIB-Luc and naïve serum-pre-injected group). Data are expressed as the means ± S.E. (PBS; n = 5, OAd-tAIB-Luc; n = 6–7).

Finally, we examined the in vivo transgene expression and antitumor effects in human tumor xenograft-bearing mice possessing the low and high titers of anti-Ad5 neutralizing antibodies (Fig. 3). The anti-Ad5 neutralizing antibody titers in the nude mice after injection with mouse anti-Ad5 serum were sufficiently higher than those in naïve serum-pre-injected mice (Table 1). The anti-Ad5 neutralizing antibody titers in the mouse serum fell within the ranges of the anti-Ad5 neutralizing antibody titers in the human serum used in this study. The anti-Ad5 neutralizing antibody titers in the immune-competent BALB/c mice receiving intravenous administration of an Ad5 vector were about fivefold higher than those in the nude mice pre-injected with a high dose of mouse anti-Ad5 serum. OAd-tAIB-Luc-mediated luciferase expression levels were significantly attenuated by pre-injection of the low- and high-dose of mouse anti-Ad5 serum, compared to those in the naïve serum-injected groups, at 3 days after OAd administration (Fig. 3B,C), indicating that pre-existing anti-Ad5 neutralizing antibodies attenuated OAd-mediated in vivo transgene expression in the tumor at an early time point.

**Table 1:** The neutralizing antibody titers following the transfer of anti-Ad5 serum.

Group	Anti-Ad5 neutralizing antibody titers*
Human serum #1	61
Human serum #2	64
Human serum #3	126
Human serum #4	283
Human serum #5	752
Human serum #6	905
Human serum #7	3802
Nude mice receiving 20 μl of naïve serum (Low)	80
Nude mice receiving 100 μl of naïve serum (High)	166
Nude mice receiving 20 μl of anti-Ad5 serum (Low)	576
Nude mice receiving 100 μl of anti-Ad5 serum (High)	1595
BALB/c mice receiving Ad5 vectors	8579

*Neutralizing anti-Ad5 antibody titers were determined by the serum dilution factor that showed a 50% reduction in luciferase production of Ad-L2 at 500 VP/cell on H1299 cells, compared with the serum-free group at 2 days after infection.

OAd-tAIB-Luc significantly suppressed the subcutaneous H1299 tumor growth, compared to that in the PBS-administered groups, in both naïve serum-pre-injected and anti-Ad5 serum-pre-injected mice (Fig. 3D). While OAd-tAIB-Luc mediated the comparable levels of tumor growth suppression in the mice receiving the low dose of anti-Ad5 serum and naïve serum, the antitumor effects of OAd-tAIB-Luc in the mice pre-injected with the high dose of anti-Ad5 serum were significantly attenuated, compared to those in naïve serum-pre-injected mice.

Next, we examined the virus genome copy numbers in the tumors on day 18 after administration. The OAd-tAIB-Luc genome copy numbers in the tumors did not significantly differ between the mice receiving the low dose of anti-Ad5 serum and naïve serum. However, the genome copy numbers of the naïve serum-pre-treated group were approximately tenfold higher than those of the mice receiving the high dose of the anti-Ad5 serum group, although there were no statistically significant differences (Fig. 3E). While the luciferase expressions in the tumor on day 3 were significantly attenuated in both low- and high-titer groups, tumor growth suppression effects and OAd genome copy numbers in the tumor on day 18 were lower only in the high-titer group than in naïve serum group. These data suggested that although antitumor effects of intratumorally administered OAd5 were significantly attenuated only in the mice possessing the high titers of anti-Ad5 neutralizing antibodies, the in vivo transgene expression at the early time point was significantly inhibited regardless of anti-Ad5 neutralizing antibody titer.

## Discussion

In this study, we examined the impact of pre-existing anti-Ad5 neutralizing antibodies on the transgene expression levels and antitumor effects of OAd-tAIB-Luc following intratumoral administration in nude mice possessing anti-Ad5 antibodies. The nude mice possessing anti-Ad5 antibodies were produced by injecting mouse anti-Ad5 serum. The titers of anti-Ad5 neutralizing antibodies in the nude mice were comparable to those of human serum used in this study (Table 1), suggesting that the nude mice in this study possessed sufficient levels of anti-Ad5 neutralizing antibodies to evaluate the inhibitory effects of anti-Ad5 neutralizing antibodies on the infection with OAd5 in the tumors.

The antitumor effects of OAds are enhanced by inserting the therapeutic gene expression cassettes in the OAd genome^5,6^; however, the impact of anti-Ad5 neutralizing antibodies on OAd5-mediated transgene expression in the tumors has remained to be elucidated. The luciferase expression and tumor cell lysis activities in the human cultured tumor cells 3 days following treatment with OAd-tAIB-Luc were attenuated in the presence of human serum, suggesting that anti-Ad5 neutralizing antibodies inhibited the infection of human cultured tumor cells with OAd-tAIB-Luc during the early time points (Figs. 1 and 2, Supplementary Fig. S2). In contrast, comparable levels of tumor cell killing activities were found after a 10-day incubation with OAd-tAIB-Luc in the presence of the low titers of anti-Ad5 antibodies and absence of anti-Ad5 antibodies (Fig. 2 and Supplementary Fig. S3). Similar phenomena were found in the mouse experiments (Fig. 3). While the luciferase expression in the tumor of mice pre-injected with a low dose of mouse anti-Ad5 serum at 3 days after OAd-tAIB-Luc administration was significantly lower than those of control mice, the tumor volumes and the Ad genome copy numbers in the tumors of mice receiving a low dose of mouse anti-Ad5 serum were similar to those of naïve serum-pre-injected mice at 18 days after OAd-tAIB-Luc injection. These were probably because certain amounts of OAd-tAIB-Luc infected the tumor cells even in the presence of anti-Ad5 antibodies, although the infection efficiencies of OAd-tAIB-Luc were lower in the presence of the low titers of anti-Ad5 antibodies than in the naïve serum-pre-injected mice. Subsequently, OAd-tAIB-Luc gradually replicated in the tumor cells, leading to efficient in vivo antitumor effects.

Although OAd-tAIB-Luc significantly suppressed the subcutaneous H1299 tumor growth in mice possessing the high titers of anti-Ad5 neutralizing antibodies, compared to PBS administration, the tumor growth suppression levels of OAd-tAIB-Luc were significantly weaker in mice possessing the high titers of anti-Ad5 neutralizing antibodies than those in the mice pre-injected with naïve serum (Fig. 3D). The Ad genome copy numbers in the tumor of mice receiving the high-dose of anti-Ad5 serum was approximately tenfold lower than those of mice pre-injected with naïve serum (Fig. 3E). The OAd-tAIB-Luc to which anti-Ad5 antibodies bound was efficiently sequestered from the tumors by macrophage uptake and/or the flow of tissue fluid, leading to significant inhibition of OAd-tAIB-Luc-mediated tumor growth suppression by anti-Ad5 antibodies.

Previous studies examined the inhibitory effects of anti-Ad5 neutralizing antibodies on the antitumor effects of OAd5 using immune-incompetent mice injected with human serum and Syrian hamsters^27–33^. However, there were disparate results regarding whether anti-Ad5 neutralizing antibodies inhibited the antitumor effects of OAd5 in tumor-bearing animal models. In addition, the impact of anti-Ad5 neutralizing antibodies on the therapeutic outcomes of OAd5 in clinical studies has not been fully evaluated. The differences in the inhibitory effects of anti-Ad5 antibodies on the antitumor effects of OAd5 among the studies were due to the differences in the experimental conditions, including the virus doses, anti-Ad5 antibody titers, administration routes of OAd5, and cancer cell types. In particular, anti-Ad5 antibody titers are considered the most crucial factor, as shown in this study. Previous studies demonstrated that anti-Ad5 neutralizing antibody titers largely affected the replication-incompetent Ad vector-mediated transgene expression levels in the tumors and cultured cells^34,35^. The anti-Ad5 neutralizing antibody titers vary widely among individuals (Table 1). Antitumor effects of OAd5 are inhibited by pre-existing anti-Ad5 neutralizing antibodies, especially when the anti-Ad5 antibody titers are high or when the anti-Ad5 antibodies can easily access OAd5 before attachment to the tumor cells. Hence, we should pay attention to patients' titers of pre-existing anti-Ad5 neutralizing antibodies.

To circumvent the inhibitory effects of anti-Ad5 neutralizing antibodies, several groups, including ours, have developed OAds based on serotypes other than Ad5, such as human Ad serotypes 3, 6, 11, 35, and non-human Ads^36–41^. The pre-existing antibody titers of these Ads are lower than those of Ad5^17–23^. In addition, the anti-Ad serotype 35 (Ad35) neutralizing antibody titers remained low after the second administration of an Ad35 vaccine vector in the phase I trial, suggesting that the neutralizing antibody induction levels differ among the Ad serotypes^42^. These OAds other than OAd5 become a superior alternative, especially when patients possess high titers of anti-Ad5 neutralizing antibodies.

Human tumor xenograft-bearing nude mice which possessed anti-Ad5 antibodies were produced by the administration of mouse anti-Ad5 serum in this study. These mice possessed sufficient levels of anti-Ad5 antibody titers; however, the effects of pre-existing anti-Ad5 immunity on the therapeutic effects and side effects of OAd5 were not completely evaluated in this mouse model. There are at least two reasons for this. First, the titers of anti-Ad5 neutralizing antibodies in the nude mice were not evaluated following OAd-tAIB-Luc administration. In clinical trials, significant and rapid elevation in titers of anti-Ad neutralizing antibodies was observed following OAd administration^43–45^. In addition, Ad protein-specific cytotoxic T cells, which are hardly induced in nude mice due to the lack of thymus, have an impact on the therapeutic effects and side effects of OAd5^46^. Therefore, further examinations will be needed to elucidate the effects of pre-existing anti-Ad5 immunity on the therapeutic and side effects of OAd5.

The nude mice receiving the naïve mouse serum showed detectable levels of anti-Ad5 neutralizing antibody titers (Table 1). It may be possible that natural antibodies and/or complements, rather than anti-Ad5 neutralizing antibodies, in the mouse serum prevented the cellular binding of Ad5, leading to a reduction in the transduction efficiencies of Ad-L2. A previous study demonstrated that natural antibodies and complements in mouse serum inhibited the cellular binding of Ad5^47^.

In conclusion, we demonstrated that OAd5-mediated transgene expression in the tumors and tumor growth suppression were significantly reduced in tumor-bearing nude mice that possessed sufficient levels of anti-Ad5 neutralizing antibodies, compared with the control mice. These results suggested that we should pay attention to the inhibitory effects of anti-Ad5 neutralizing antibodies on OAd5-mediated transgene expression and antitumor effects.

## Materials and methods

### Cells and reagents

HeLa (a human cervical carcinoma cell line) and PANC-1 (a human pancreatic adenocarcinoma cell line) cells were cultured in Dulbecco’s modified Eagle’s medium supplemented with 10% fetal bovine serum (FBS) and antibiotics. H1299 (a non-small cell lung carcinoma cell line) cells were cultured in RPMI1640 supplemented with 10% FBS and antibiotics. Three batches of Japanese human serum (#1, #2, #4) and four batches of human True A serum (#3, #5, #6, #7) were purchased from KAC Co. Ltd (Kyoto, Japan). HeLa cells were obtained from the JCRB Cell Bank (Tokyo, Japan). PANC-1 and H1299 cells were purchased from the American Type Culture Collection (ATCC; Manassas, VA).

### Viruses

The CMV promoter-driven firefly luciferase-expressing replication-incompetent Ad5 vector, Ad-L2, was previously produced^48^. The recombinant OAd5 expressing an hTERT promoter-driven Ad5 E1 gene and CMV promoter-driven firefly luciferase gene (OAd-tAIB-Luc) was previously constructed^25^. The determination of virus particle (VP) titers was accomplished according to Maizel et al*.*^49^

### In vitro luciferase assay

Tumor cells were seeded on a 96-well plate at 0.8–2.0 × 10^4^ cells/well. On the following day, Ad-L2 and OAd-tAIB-Luc were pre-incubated with mouse and human serum for 30 min at room temperature, followed by addition to tumor cells at 200 VP/cell. After a 3-day incubation, luciferase activity in the cells was determined using the Bright-Glo Luciferase Assay System (Promega, Madison, WI).

### Measurement of cell viability

Tumor cells were seeded on a 96-well plate at 0.8–2.0 × 10^4^ cells/well. On the following day, OAd-tAIB-Luc was pre-incubated with mouse and human serum for 30 min at room temperature, followed by infection of tumor cells at 200 VP/cell. Cell viabilities were determined using a Cell Counting Kit-8 (Dojindo Laboratories, Kumamoto, Japan) on the indicated days according to the manufacturer’s protocol.

### Preparation of mouse serum

A replication-incompetent Ad5 vector, Ad-null^50^, was intravenously administered to BALB/c mice (Nippon SLC, Hamamatsu, Japan) at a dose of 1.0 × 10^10^ VP/mouse, followed by re-administration 14 days after the first administration. After 24-days from the first injection, blood samples were collected via inferior vena cava. Following a 30-min incubation at room temperature, blood samples were incubated overnight at 4 °C. The supernatants were collected as a mouse anti-Ad5 serum after centrifugation at 7000 rpm for 15 min. Naïve serum samples were similarly recovered from naïve mice.

For the serum collection from nude mice pre-injected with mouse anti-Ad5 serum, the blood samples were taken via inferior vena cava an hour after intravenous administration of mouse anti-Ad5 serum. The serum was isolated from the blood samples as described above.

### Determination of anti-Ad5 neutralizing antibody titers

H1299 cells were seeded on a 96-well plate at 8.0 × 10^3^ cells/well. On the following day, Ad-L2 was pre-incubated with diluted serum for 30 min and added to H1299 cells at 500 VP/cell. Luciferase activity was determined 2 days after transduction. The neutralizing antibody titers were defined as the serum dilution factor that showed a 50% reduction in luciferase production compared with the serum-free group.

### OAd-mediated growth inhibition of subcutaneous tumor xenografts in mice

H1299 cells (3.0 × 10^6^ cells per mouse) mixed with matrigel (Corning, Corning, NY) were subcutaneously injected into the right flank of 5-week-old female BALB/c nu/nu mice (Nippon SLC). Mice were randomly assigned to four groups when the tumors grew to approximately 8–12 mm in longer dimensions. Mouse anti-Ad5 and naïve serum were administered at 20 μl/mouse (low titer) or 100 μl/mouse (high titer) via the tail vein. PBS and OAd-tAIB-Luc were intratumorally administered at a dose of 2.0 × 10^9^ VP/mouse 1 h after serum injection. The tumor size was measured every 3 day using vernier calipers. The following formula calculated tumor volume: tumor volume (mm^3^) = *a* × *b*^2^ × 3.14 × 6^–1^, where *a* is the longest dimension, and *b* is the shortest. D-luciferin potassium salt (FUJIFILM Wako Pure Chemical Corporation, Osaka, Japan) in PBS was intravenously injected into the tumor-bearing mice via the tail vein at a dose of 2.0 mg/mouse 3 days after virus administration. Luciferase expression was monitored and analyzed using NightOWL LB983 (BERTHOLD Technologies, Tokyo, Japan).

### Determination of Ad genome copy numbers in the tumors

Total DNA of tumor tissues was isolated using DNAzol (Molecular Research Center, Cincinnati, OH) 18 days after virus administration. The OAd-tAIB-Luc genome copy numbers in the tumor tissues were quantified by real-time PCR analysis using THUNDERBIRD Next SYBR qPCR Mix reagents (TOYOBO, Osaka, Japan) and a StepOnePlus System (Thermo Fisher Scientific). The sequences of the primers are the following: Forward, 5’-GGGATCGTCTACCTCCTTTTG-3’, Reverse, 5’-GGGCAGCAGCGGATGAT-3’.

### Statistical analyses

One-way analysis of variance (ANOVA) with Dunnett's multiple comparisons post hoc test and two-way ANOVA followed by Bonferroni's multiple comparisons test were performed using GraphPad Prism version 9.0 (GraphPad Software, San Diego, CA). Data are presented as means ± S.D. or S.E.

### Ethics declarations

The Animal Experiment Committee of Osaka University approved animal experiments in this study. We performed all experiments in accordance with the relevant regulations and guidelines, including the ARRIVE guidelines.

## Supplementary Information

Supplementary Information.

## Data Availability

All data generated or analyzed during this study are included in this published article (and its Supplementary Information files).
