# Supplementary material for: Effects of pre-existing anti-adenovirus antibodies on transgene expression levels and therapeutic efficacies of arming oncolytic adenovirus
Source: Sci Rep. 2022 Dec 13;12:21560. doi: 10.1038/s41598-022-26030-3 (PMC9747716; doi:10.1038/s41598-022-26030-3)

Supplementary information

Tittle:

Effects of pre-existing anti-adenovirus antibodies on transgene expression levels and therapeutic efficacies of arming oncolytic adenovirus

Author list:

Ryosuke Ono,[1] Fumitaka Nishimae,[1] Takuro Wakida,[1] Fuminori Sakurai,[1,*] and Hiroyuki Mizuguchi[1,2,3,4,5,*]

[1] Laboratory of Biochemistry and Molecular Biology, Graduate School of Pharmaceutical Sciences, Osaka University, Osaka, Japan

[2] Laboratory of Functional Organoid for Drug Discovery, Center for Drug Discovery Resources Research, National Institutes of Biomedical Innovation, Health and Nutrition, Osaka, Japan

[3] Global Center for Medical Engineering and Informatics, Osaka University, Osaka, Japan

[4] Integrated Frontier Research for Medical Science Division, Institute for Open and Transdisciplinary Research Initiatives (OTRI), Osaka University, Osaka, Japan

[5] Center for Infectious Disease Education and Research (CiDER), Osaka University, Osaka, Japan

Supplementary figure S1.

The luciferase expression levels in human cultured cells following treatment with Ad-L2 and OAd-tAIB-Luc

A) H1299 B) HeLa C) PANC1 cells were transduced with Ad-L2 and OAd-tAIB-Luc at 200 VP/cell. The luminescence (relative light unit) was measured on day 3. These data of luminescence were used as a control and normalized to 100% in Fig. 1. These data are expressed as the means ± SD. (n=4).

Supplementary figure S1

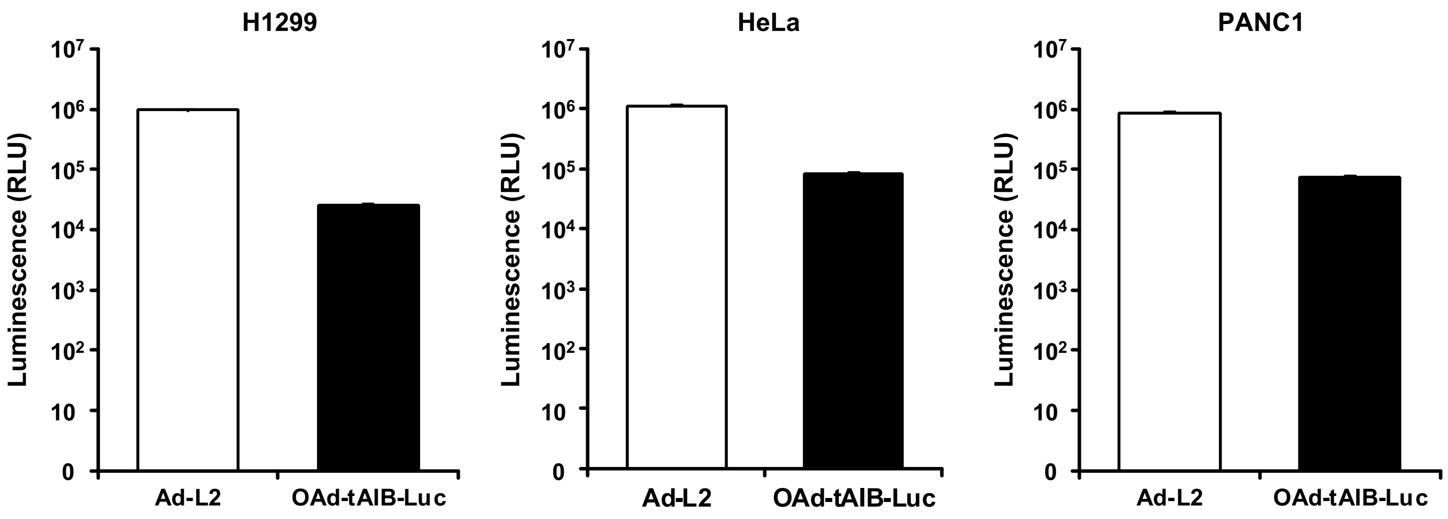

Supplementary figure S2.

Cell viabilities on day 3 following treatment with OAd-tAIB-Luc.

A) H1299 B) HeLa C) PANC1 cells were treated with OAd-tAIB-Luc at 200 VP/cell. Cell viabilities were determined on day 3. The cell viabilities of mock-treated cells were normalized to 100%. These data are expressed as the means ± SD. (n=4) and analyzed by one-way ANOVA followed by Dunnett's multiple comparisons test. *; $P<0.05$, **; $P<0.01$, ***; $P<0.001$, n.s.; not significant = $P>0.05$. (vs. No serum group).

Supplementary figure S2

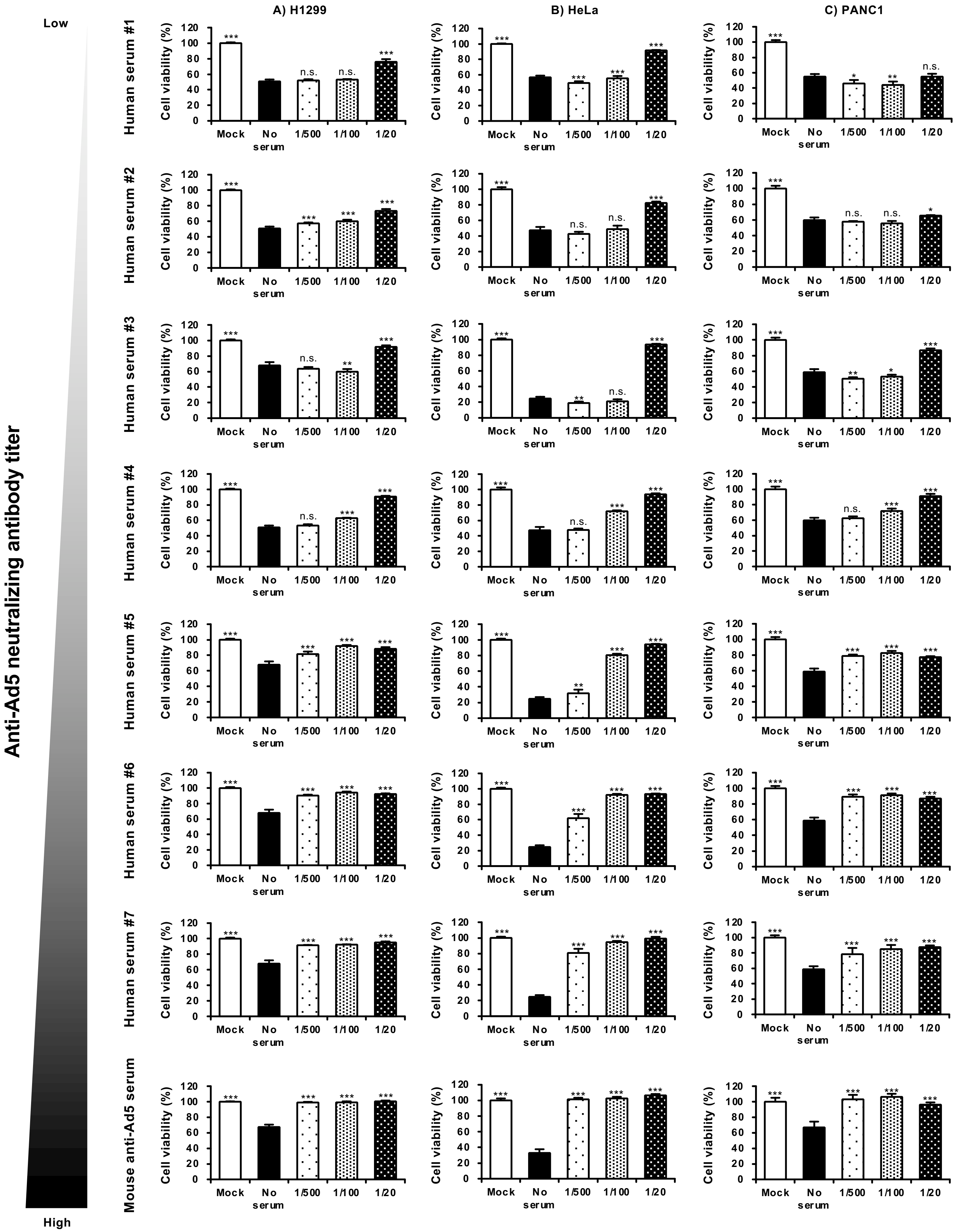

Supplementary figure S3.

Cell viabilities on day 10 following treatment with OAd-tAIB-Luc.

A) H1299 B) HeLa C) PANC1 cells were treated with OAd-tAIB-Luc at 200 VP/cell. Cell viabilities were determined on day 10. The cell viabilities of mock-treated cells were normalized to 100%. These data are expressed as the means ± SD. (n=4) and analyzed by one-way ANOVA followed by Dunnett's multiple comparisons test. *; $P<0.05$, **; $P<0.01$, ***; $P<0.001$, n.s.; not significant = $P>0.05$. (vs. No serum group).

Supplementary figure S3

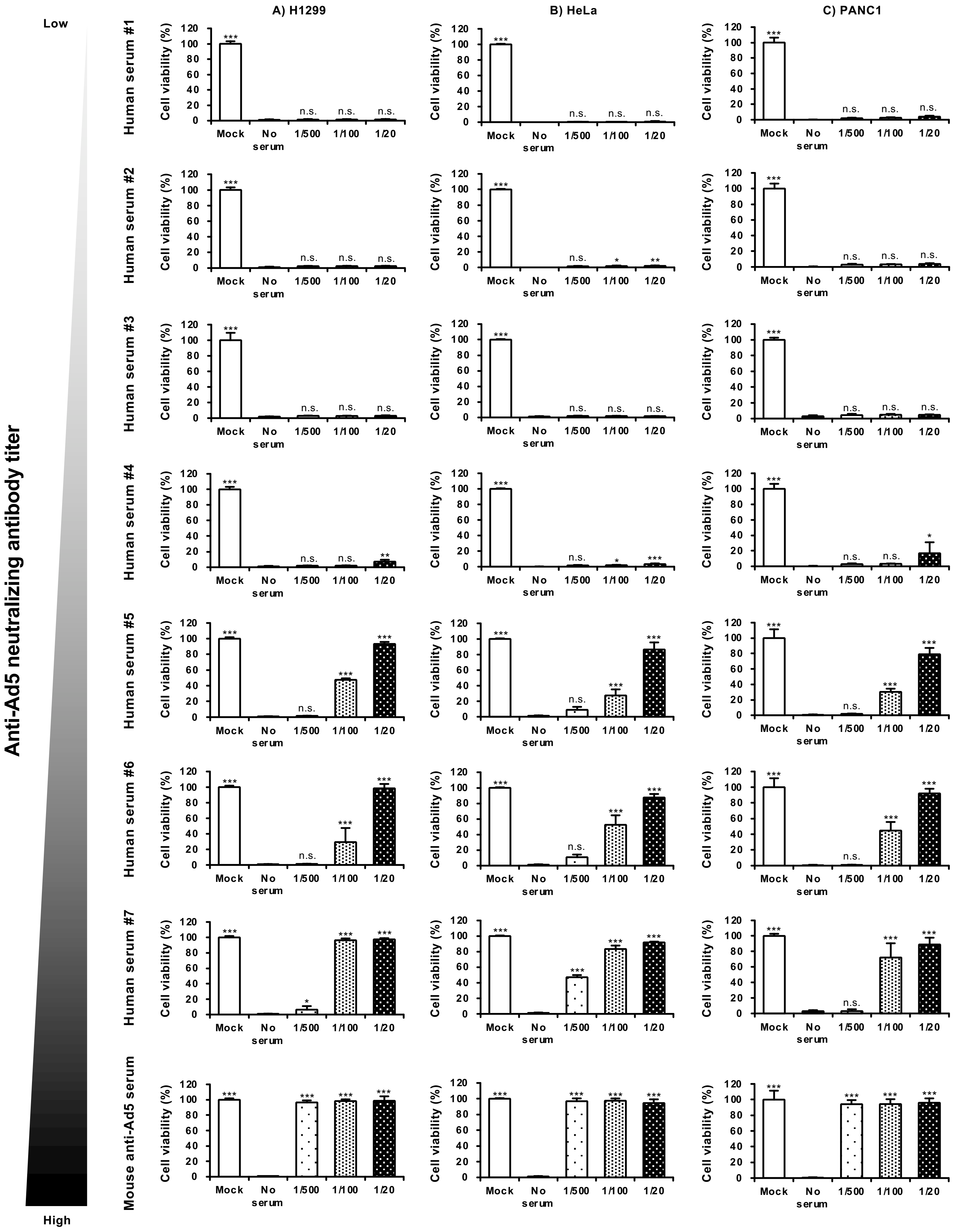

Supplement: Supplementary file 1 — Supplementary Information. [file 41598_2022_26030_MOESM1_ESM.pdf]
